# Management of Failed Apexification Associated with Internal Root Resorption and Apical Calcification: A 56-Month Case Report

**DOI:** 10.3390/dj14070453

**Published:** 2026-07-20

**Authors:** Yuzhuo Wang, Shujun Ran

**Affiliations:** 1 Department of Endodontics and Operative Dentistry, Shanghai Ninth People’s Hospital, College of Stomatology, Shanghai Jiao Tong University School of Medicine, Shanghai 200011, China; wyz19834671279@163.com; 2 Department of Endodontics and Operative Dentistry, Shanghai Ninth People’s Hospital, College of Stomatology, Shanghai Jiao Tong University School of Medicine, National Center for Stomatology, National Clinical Research Center for Oral Disease, Shanghai Key Laboratory of Stomatology, Shanghai 200011, China

**Keywords:** apical barrier technique, internal root resorption, Er:YAG laser, iRoot BP, root reinforcement, long-term follow-up, endodontic treatment

## Abstract

**Objective**: To report the conservative management of a complex failed apexification in a permanent premolar complicated by severe internal root resorption, irregular apical calcification, and exceptionally thin root canal walls and to evaluate its long-term (56-month) clinical and radiographic outcomes. **Methods**: A 27-year-old male presented with chronic apical periodontitis in the right mandibular first premolar. A previous apexification attempt had failed, resulting in arrested root development, an apical calcified bridge, and extensive internal root resorption. CBCT imaging revealed a critical root canal wall thickness of less than 1 mm in the middle and coronal thirds. To avoid extraction or invasive surgery, a non-surgical apical barrier technique was performed. Due to the severe structural compromise and high risk of root fracture, mechanical instrumentation was minimized; instead, chemomechanical debridement was optimized using Er:YAG laser-activated irrigation. The apical region was sealed with a bioceramic material (iRoot BP), and the weakened root structure was reinforced intraradicularly with dual-cure flowable resin, followed by progressive occlusal adjustments. **Results**: At the 3-month follow-up, the patient was completely asymptomatic, and radiographs indicated initial periapical bone healing. The 56-month follow-up demonstrated progressive formation of mineralized tissue, apparent thickening of the root canal walls at root apex, complete resolution of periapical radiolucency, and a stable, functional occlusion. **Conclusions**: This case demonstrates that teeth with severe structural compromise and internal resorption following failed apexification are not necessarily hopeless. A tailored conservative approach, integrating advanced laser-assisted disinfection, bioceramic apical barriers, internal resin reinforcement, and meticulous occlusal management, can yield highly successful long-term outcomes, ensuring the preservation of the natural dentition.

## 1. Introduction

Dens evaginatus is a developmental anomaly primarily affecting mandibular premolars in which an accessory cusp fractures soon after eruption, leading to pulp necrosis and arrested root development [1,2]. The resulting immature tooth presents with an open apex and thin, fragile dentinal walls—conditions that make both debridement and apical sealing technically demanding [3]. Apexification was historically the standard management approach, aiming to induce a calcified apical barrier using long-term calcium hydroxide (Ca(OH)_2_) [4]. However, Ca(OH)_2_ apexification carries well-documented drawbacks: a lengthy treatment span of 6–24 months with a reported failure rate of up to 30%, a high reliance on patient compliance across multiple visits, and critically, the weakening of radicular dentin due to prolonged Ca(OH)_2_ application, which predisposes the tooth to fracture [5,6]. Retrospective data indicate that up to 32% of teeth treated with Ca(OH)_2_ apexification ultimately sustain root fractures [7]. Even when macroscopic barrier formation appears radiographically successful, residual necrotic tissue within apical irregularities can sustain chronic infection, driving inflammatory internal root resorption (IRR) and periapical destruction [3,8].

Management of failed apexification remains challenging because persistent infection and structural compromise may coexist [9]. Current nonsurgical treatment strategies mainly include regenerative endodontic procedures (REPs) and apical barrier techniques using calcium silicate-based materials (CSCs) [10,11]. REPs aim to revascularize the canal and promote continued root maturation through stem cell-mediated tissue regeneration. However, REP success is strongly tied to patient age, apical foramen diameter, and viable progenitor cell populations in the periapical region, all of which are substantially diminished after years of chronic infection [11,12]. Moreover, REP carries a 14–19% failure rate attributable to persistent infection, and its long-term outcomes in the adult patient remain insufficiently evidenced [13]. In contrast, the bioceramic apical barrier technique, using CSCs such as iRoot BP Plus, provides an immediate, infection-independent apical seal with documented 6-year clinical success rates in adult permanent teeth [14]. However, unlike REPs, the apical barrier technique does not promote continued root maturation or dentinal wall thickening [15]. Consequently, the structurally compromised immature root remains vulnerable to fracture, which may adversely affect its long-term prognosis [11].

The clinical challenge deepens considerably in cases of failed apexification presenting to the adult patient years or decades later. At this stage, the original pathology has often evolved into a compound problem: concurrent active infection, progressive IRR, ectopic calcification within the root canal, and critically compromised residual wall thickness. In current clinical practice, extraction is often considered the treatment of choice for such cases, as no standardized tooth-preserving strategy has yet been established.

In the present report, we describe the conservative management of a failed apexification case complicated by IRR, irregular apical calcification, and severe structural compromise. Treatment was accomplished through a nonsurgical approach using a calcium silicate-based apical barrier combined with intracanal reinforcement and long-term occlusal management, resulting in favorable clinical and radiographic outcomes after 56 months of follow-up. This case highlights the potential for preserving severely compromised immature teeth and may provide a practical reference for the management of similar challenging cases for which standardized tooth-preserving strategies have not yet been established.

## 2. Case Report

### 2.1. Patient Information

This case is reported in line with the CARE guidelines [16]. Written informed consent was obtained from the patient for publication of the case details and accompanying clinical images. The patient had no active systemic diseases or medications, no allergy to drugs or local anesthesia, and did not smoke.

### 2.2. Clinical and Radiographic Examination

A 27-year-old Chinese male was referred to the Department of Endodontics with a chief complaint of swelling and pain in the right mandibular posterior region persisting for over one month. Medical history was non-contributory. He reported that more than 10 years prior, the same tooth had undergone pulp therapy and apexification following an episode of acute dental pain. There was no history of dental trauma, recent temperature sensitivity, nocturnal pain, or biting pain.

**Figure 1:**
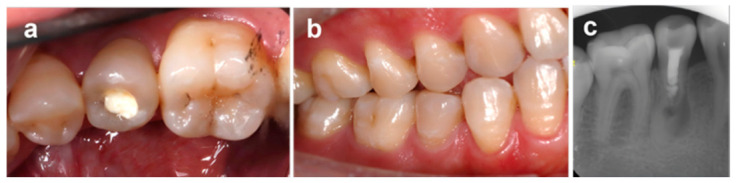
Initial clinical examination. (**a**) Intraoral photograph showing the occlusal white filling on tooth #44. (**b**) Intraoral occlusal photograph illustrating posterior crossbite and anterior edge-to-edge relationship. (**c**) Periapical radiograph of tooth #44 demonstrating root canal filling, IRR radiolucency, and periapical changes.

Intraoral examination revealed a white coronal filling on the occlusal surface of the mandibular right first premolar (tooth #44, according to the FDI World Dental Federation notation), with no detectable secondary caries (Figure 1a). Percussion tenderness was absent and physiological tooth mobility was preserved. The buccal gingiva in the apical region appeared erythematous and edematous, with a probing depth of 2 mm and no sinus tract visible. The patient exhibited a right posterior crossbite, anterior edge-to-edge occlusal relationship, and mild dental crowding, producing abnormal buccal lateral forces on tooth #44 during mandibular excursions (Figure 1b). Oral hygiene was adequate (calculus index 0–1; plaque index 0–1).

Periapical radiography (Figure 1c) demonstrated:

1. Radiopaque filling material within the root canal;
2. Incomplete adaptation of filling material to canal walls at the middle root third;
3. An oval-shaped radiolucency spanning the middle-to-apical root thirds, consistent with IRR;
4. Absence of a visible root canal in the apical segment;
5. A periapical radiolucency on the mesial aspect of the middle-to-apical root third.

**Figure 2:**
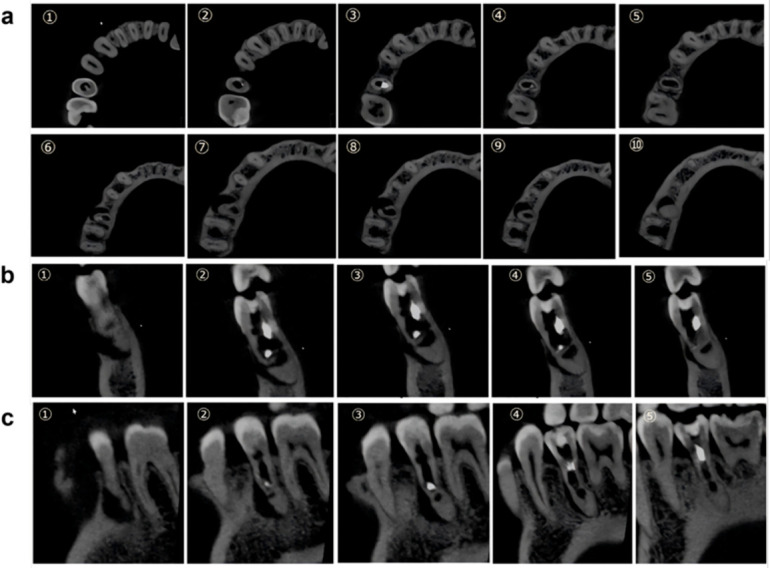
Pre-treatment CBCT images (filling material removed prior to imaging). (**a**) Cross-sectional views arranged sequentially from coronal to apical direction (images ①–⑩). (**b**) Coronal views arranged sequentially from mesial to distal direction (images ①–⑤). (**c**) Sagittal views arranged sequentially from buccal to lingual direction (images ①–⑤), illustrating the buccally positioned calcified bridge, IRR lacunae, thin walls, and periapical communication.

Because conventional radiography was insufficient to characterize the three-dimensional anatomy, cone-beam computed tomography (CBCT) was performed after removal of the radiopaque filling (to minimize scatter artifact). CBCT imaging (Figure 2) revealed:

1. Irregular resorption lacunae in the coronal and middle root canal thirds;
2. Critically thin residual root canal walls (<1 mm at several locations);
3. A calcified bridge formation eccentrically positioned on the buccal aspect of the apical segment;
4. Open communication between the root canal space below the calcified bridge and the buccal alveolar bone;
5. Complete calcification of the apical segment with no discernible root canal lumen.

### 2.3. Diagnosis and Treatment Planning

Clinical diagnoses for tooth #44 were established as:

- History of previous endodontic treatment;
- Symptomatic chronic apical periodontitis;
- Incomplete root development secondary to failed apexification;
- Inflammatory IRR.

Given the patient’s age (27 years), the long-standing chronicity of periapical infection (>10 years), the severely compromised apical anatomy, and the patient’s expressed preference for tooth preservation, the apical barrier technique using iRoot BP Plus was selected as the definitive treatment modality. Regenerative endodontic procedures (REPs) were considered but deemed less suitable given the patient’s age, the extent of periapical destruction limiting viable stem cell populations, and the ovoid periapical radiolucency. Microsurgical apicoectomy was designated as a contingency intervention if the non-surgical approach failed. Tooth extraction was discussed with the patient, who declined and provided written informed consent for the proposed treatment plan.

### 2.4. Procedure

#### 2.4.1. Debridement and Intracanal Medication

**Figure 3:**
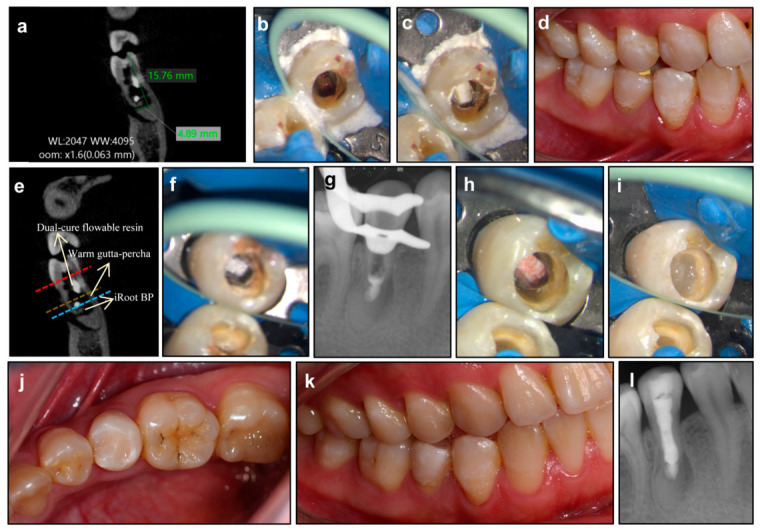
Treatment procedure. (**a**) CBCT working length verification at 15.8 mm. (**b**) Er:YAG laser-activated irrigation for root canal disinfection. (**c**) Calcium hydroxide intracanal medicament placement. (**d**) Resolution of gingival inflammation at the second visit. (**e**) Schematic diagram of layered root canal filling materials. (**f**) iRoot BP Plus filling the apical segment via the lingual pathway. (**g**) Radiograph confirming iRoot BP Plus placement. (**h**) Warm gutta-percha backfill over iRoot BP Plus. (**i**) Dual-cure flowable composite resin reinforcing the middle and coronal canal. (**j**) Coronal composite resin restoration. (**k**) Occlusal adjustment. (**l**) Final post-treatment radiograph.

Rubber dam isolation (OptiDam, Kerr Dental, Brea, CA, USA) was achieved with supplemental gingival barrier placement (OraSeal, Ultradent Products Inc., South Jordan, UT, USA). Under a dental operating microscope (OMS3500, ZUMAX, Suzhou, China), the existing coronal restoration and root canal filling material were removed using ultrasonic instrumentation (ET18D, SATELEC, Mérignac, France). Granulation tissue was confirmed in the apical root canal portion. An #80 K-file (MANI^®^ K-FILES, MANI, Inc., Utsunomiya, Japan) was used for granulation tissue removal while deliberately preserving the calcified apical bridge. Working length was verified with CBCT measurement at 15.8 mm, confirming that the measurement extended beyond the eccentrically positioned calcified bridge to the level of the open periapical communication via the patent lingual pathway (Figure 3a). Chemo-mechanical debridement consisted of alternating irrigation with 1% NaClO and 17% EDTA (MD-ChelCream, META BIOMED Co., Ltd., Cheongju-si, Republic of Korea), followed by erbium laser-activated irrigation (Er:YAG; Figure 3b). Er:YAG laser (Fotona SWEEPS™, Fotona d.o.o., Ljubljana, Slovenia) was activated at settings of 10–20 mJ, 15 Hz, and ~0.3 W, with a HC14 N handpiece and a SWEEPS400/14 flat-tip fiber immersed in 1% NaOCl. Each cycle comprised 30 s of laser activation and 30 s of pause; the number of cycles (up to three) was determined by the observed canal cleanliness. The Er:YAG laser (wavelength 2940 nm) exhibits a high affinity for water, generating photomechanical cavitation that enhances irrigant activation within anatomically inaccessible areas of the root canal system. This mechanism facilitates biofilm disruption and smear layer removal while producing minimal thermal effects on the dentinal walls, making it particularly suitable for structurally compromised teeth with thin root walls [17,18]. After irrigating with sterile saline, the root canal was dried using sterile paper points (size #40, 0.06 taper) inserted sequentially until the final paper point remained completely dry and free of any moisture or blood staining. Finally, the canal was filled with Ca(OH)_2_ paste (ApexCal, Ivoclar Vivadent AG, Schaan, Liechtenstein) (Figure 3c). The tooth was temporized with Cavit (3M ESPE AG, Seefeld, Germany).

#### 2.4.2. Apical Barrier Placement and Obturation

One week later, gingival erythema and edema had resolved (Figure 3d), and percussion sensitivity was absent. After re-isolation, temporary filling removal, and sonic-activated irrigation to remove Ca(OH)_2_, the canal was dried and confirmed free of significant bleeding. iRoot BP Plus (Innovative Bioceramix Inc., Vancouver, BC, Canada), a premixed bioceramic calcium silicate cement, was delivered via the lingual pathway of the calcified bridge using a dedicated carrier and condensed below and covered the calcified bridge to establish a contiguous and solid apical plug (Figure 3f,g). Radiographic verification confirmed the appropriate position of the apical barrier material and a plug thickness of approximately 4–5 mm. Adequate placement was confirmed by the following criteria: (1) The material was condensed firmly against the calcified bridge/apical stop with definite resistance; (2) Radiographic verification—a periapical radiograph was taken immediately after placement to confirm that the material was condensed below and around the calcified bridge without voids, and that the plug extended continuously from the apical aspect to at least 4 mm. The coronal margin of the iRoot BP Plus plug was covered with 1 mm warm gutta-percha (MaxFill-G, REFINE Co., Ltd., Yokohama, Japan) to create a barrier layer (Figure 3h). Considering the critically thin root canal walls in the middle and coronal segments, which precluded aggressive mechanical instrumentation and rendered the root vulnerable to fracture, these segments were reinforced with dual-cure flowable composite resin (3M Filtek, 3M ESPE AG, Seefeld, Germany) to a depth of approximately 10 mm from the apex (Figure 3i). The coronal 5 mm was restored with posterior composite resin (3M P60, 3M ESPE AG, Seefeld, Germany) (Figure 3j). Occlusal adjustments were initiated to reduce lateral forces on the buccal cusp (Figure 3k), and a final radiograph confirmed the completed restoration (Figure 3l).

### 2.5. Follow-Up

**Figure 4:**
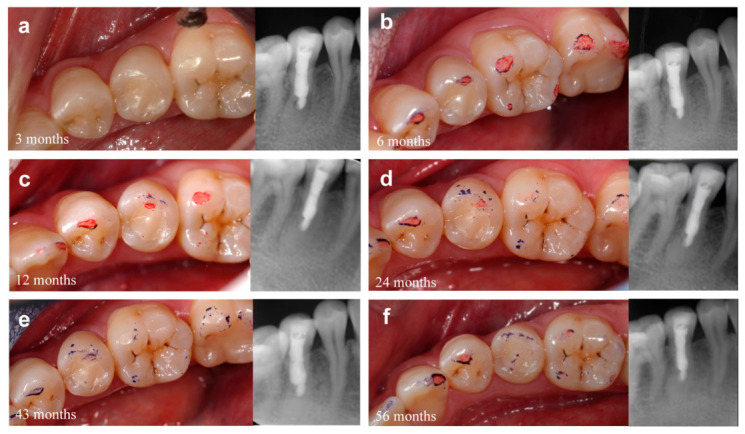
Follow-up clinical photographs and periapical radiographs. (**a**) 3-month follow-up: periapical bone density improvement and continuous PDL space. (**b**) 6-month follow-up: light occlusal contact adjustment. (**c**) 12-month follow-up: occlusal contact redirected onto composite resin. (**d**) 24-month follow-up: uniform multi-point occlusal contact. (**e**) 43-month follow-up: stable occlusion and continued bone regeneration. (**f**) 56-month follow-up: favorable long-term outcome with no radiographic or clinical signs of pathology.

The patient was evaluated clinically and radiographically at 3, 6, 12, 24, 43, and 56 months post-treatment. At 3 months, the patient was asymptomatic; periapical radiography demonstrated increased bone density in the periapical region and a continuous periodontal ligament space around tooth #44 (Figure 4a). Subsequent follow-ups confirmed a consistent positive trend: progressive periapical bone formation, radiographic evidence of root canal wall thickening (indicative of hard tissue apposition), and complete absence of symptoms or clinical signs of reinfection.

Given the severe structural compromise of the tooth and the presence of a posterior crossbite, a progressive, multi-step occlusal adjustment protocol was implemented to prevent root fracture. A total of four adjustments were performed over the initial 24-month period. Immediately following the definitive restoration, lateral excursive interferences on the buccal cusp were eliminated to reduce abnormal shear forces. At the 6-month recall, the occlusion was adjusted to maintain only light contact in maximum intercuspation to protect the healing periodontium (Figure 4b). At 12 months, as periapical healing progressed, adjustments were specifically refined to direct occlusal forces onto the composite resin restoration, thereby redistributing stress away from the fragile dentinal walls (Figure 4c). By 24 months, with robust bone regeneration confirmed, uniform multi-point occlusal contacts were established across the tooth and restoration (Figure 4d). This tailored functional loading was well-tolerated, and occlusal stability was successfully maintained at both the 43-month (Figure 4e) and 56-month (Figure 4f) follow-ups, with no further adjustments required.

## 3. Discussion

### 3.1. Etiological Considerations and Therapeutic Decision-Making

The present case most likely originated from complications associated with dens evaginatus, which is a well-recognized developmental anomaly affecting mandibular premolars. Although the initial episode occurred more than 10 years before presentation and could not be retrospectively verified, the combination of the affected tooth, arrested root development following apexification, and the patient’s clinical history strongly suggests pulpal necrosis secondary to fracture of an accessory cusp.

Regenerative endodontic procedures (REPs) depend on three interdependent biological prerequisites: an adequate apical foramen diameter (typically ≥0.5 mm) to permit vascular ingrowth, viable stem cells from the apical papilla (SCAP), and a sterile scaffold environment [19,20]. In this 27-year-old patient, all three were compromised. Concerning stem cell viability, studies have shown that the regenerative potential of SCAP diminishes substantially with patient age and is further reduced by prolonged periapical infection; low-grade chronic inflammation and its attendant oxidative microenvironment are toxic to dental progenitor cells, reducing their proliferative and differentiative capacity [21,22]. In this patient, over a decade of periapical infection had almost certainly depleted the residual SCAP population in the apical region. Concerning the apical anatomy, the eccentric buccal calcified bridge and complete obliteration of the apical foramen made it anatomically impossible to reliably induce the periapical hemorrhage needed to form a regenerative blood clot scaffold [19,20]. Furthermore, intracanal calcification itself is a recognized complication of REPs (occurring in up to 43% of cases according to recent long-term cohort data [5]), meaning that even a successfully initiated REP in this tooth could have perpetuated the existing calcification problem [23]. For these reasons, a REP was dismissed as anatomically infeasible and biologically unpredictable in this case.

### 3.2. Selection of Apical Barrier Materials

The bioceramic apical barrier technique was selected because it bypasses the need for regenerative biology: it provides an immediate artificial apical barrier after adequate disinfection without relying on stem cell recruitment or vascular ingrowth. Among the available CSCs, iRoot BP Plus was chosen on the basis of three convergent properties. First, its premixed, injectable consistency allows for reliable delivery into the narrow, irregularly shaped apical space distal to the calcified bridge via the lingual pathway, without the mixing variability that affects MTA [24,25]. Second, iRoot BP Plus sets in the presence of moisture through a hydraulic reaction, which is particularly valuable in a canal environment where complete desiccation could not be guaranteed given the proximity of the resorption lacunae to the periapical communication [14]. Third, and most clinically significant for this case, iRoot BP Plus exhibits documented bioactivity: its ion release profile promotes the upregulation of mineralization-related genes in periapical cells and enhances SCAP migration [26]. This property may explain the progressive root canal wall thickening and periapical bone regeneration observed radiographically over the 56-month follow-up. Given the long-standing chronic infection, extensive internal root resorption, and the likely destruction of the original odontoblast layer, true tubular dentin regeneration appears unlikely. Instead, the newly formed mineralized tissue may represent cementum-like or osteodentin-like tissue deposited by periodontal ligament-derived or periapical mesenchymal cells following infection control and the establishment of an adequate apical seal. The sustained release of calcium ions from iRoot BP Plus may further promote mesenchymal cell differentiation and mineralized tissue deposition in the apical region [24,26].

Even with a successfully placed apical seal, the long-term survival of a tooth with sub-millimeter root canal walls depends on two additional imperatives: achieving thorough disinfection without further weakening the walls, and subsequently reinforcing the tooth against fracture under functional load.

### 3.3. Infection Control and Maintenance of Long-Term Structural Integrity

With respect to disinfection, the extreme thinness of the canal walls (<1 mm) precluded all rotary mechanical instrumentation, rendering the case almost entirely chemo-mechanical. Conventional needle irrigation, while effective on accessible canal surfaces, leaves residual biofilm in root canal irregularities, lateral canals, and dentinal tubules—areas that were particularly abundant in this case given the IRR lacunae [27]. Er:YAG laser-activated irrigation (PIPS/SWEEPS modalities) was therefore employed as the primary disinfection adjunct. A 2025 systematic review confirmed that Er:YAG laser activation generates photoacoustic streaming and cavitation phenomena that disrupt multispecies biofilms in anatomically inaccessible zones, surpassing conventional irrigation modalities in this regard, with minimal thermal risk to dentinal walls [28]. A 2024 in vitro study using SEM and CLSM further demonstrated that both PIPS and SWEEPS modalities of Er:YAG activation achieved significant biofilm reduction in simulated apical grooves and dentinal tubules, which is the precise anatomical scenario encountered in this case [29]. Together with sequential 1% NaClO and 17% EDTA irrigation [27], Er:YAG activation provided high confidence in adequate disinfection within the constraints imposed by the structurally compromised root.

Passive ultrasonic irrigation (PUI) is highly effective at biofilm disruption; however, the rigid metal tips commonly used in PUI carry a risk of inadvertent dentin gouging upon file-to-wall contact, which could be detrimental in roots with critically thin walls and calcific bridge (<1 mm) [30]. Similarly, mechanical agitation tools like endo-brushes rely on physical friction to remove biofilms and may be less effective at penetrating deep internal resorption lacunae [31]. Conversely, sonic activation systems (such as the EndoActivator or EDDY) present a highly viable alternative. These devices utilize flexible, non-cutting polymer or polyamide tips that generate robust acoustic microstreaming without the risk of removing additional dentin or causing micro-fractures upon wall contact [32,33]. Therefore, sonic activation is also an alternative method for adjunctive chemical preparation for structurally vulnerable teeth.

With respect to the definitive coronal restoration, preservation of the remaining tooth structure was prioritized. In the present case, four coronal walls remained intact after removal of the defective restoration, making full-coverage crown preparation unnecessary. Current evidence suggests that direct adhesive restoration is an appropriate approach for endodontically treated teeth with sufficient residual coronal structure, whereas crowns are generally indicated for teeth with more extensive tissue loss [18]. Likewise, fiber post placement was not considered necessary. In the absence of an adequate ferrule, fatigue studies have shown that fiber posts provide limited improvement in long-term survival and may introduce additional interfaces susceptible to microleakage, whereas composite-core restorations tend to exhibit more favorable and repairable failure patterns [34]. More importantly, this tooth exhibited critically thin middle-root dentinal walls secondary to long-standing internal root resorption and failed apexification. Therefore, dual-cure flowable composite resin was extended into the middle and coronal canal thirds to provide intracanal reinforcement while preserving the remaining sound tooth structure. Biomechanical evidence from studies of thin-walled endodontically treated teeth consistently demonstrates that intracoronal bonding of dual-cure composite resin to root canal walls substantially increases the fracture resistance. The bonded resin mass acts as an intraradicular reinforcing structure, distributing lateral stress across the bonded interface rather than concentrating it at the thinnest point of the root [23,35]. Combined with careful occlusal management, this minimally invasive adhesive strategy contributed to satisfactory clinical function and radiographic stability throughout the 56-month follow-up period.

Long-term occlusal management was also considered essential in this case. Because the patient presented with a posterior crossbite, unfavorable lateral forces could have increased stress concentration within the weakened root and adhesive complex. Therefore, periodic occlusal evaluation and selective adjustment were performed to eliminate premature contacts and reduce functional loading on the restored tooth. The favorable outcome over the 56-month follow-up highlights the importance of continuous functional maintenance in the long-term preservation of structurally compromised teeth.

Faced with the radiographic and clinical presentation of this tooth, extraction might reasonably have been considered the path of least resistance. However, current evidence firmly supports natural tooth preservation over extraction and implant replacement wherever feasible, given the advantages of the natural periodontal ligament in proprioception, alveolar bone maintenance, and long-term oral health [36,37]. For adult patients with a premolar tooth retaining intact coronal walls and a restorable crown, direct composite resin restoration, rather than full-coverage crown placement, is also consistent with evidence-based minimally invasive principles, avoiding the unnecessary sacrifice of sound tooth structure [18].

### 3.4. Clinical Implications and Limitations

The 56-month radiographic follow-up in this case—including progressive periapical bone regeneration, cessation of resorptive activity, and obvious morphological changes in the root canal wall—suggests that under strictly selected indications, even teeth presenting with multiple concurrent complex lesions may retain the potential for long-term healing. This observation implies that a biologically oriented conservative treatment strategy may offer clinical reference value in complex endodontic failure cases; however, further studies are warranted to validate its generalizability.

The present case report has favorable long-term healing results while several limitations should be acknowledged. First, as a single-case observation, the clinical outcomes cannot be generalized to all similar presentations of failed apexification, and should therefore be interpreted with caution. Second, cone-beam computed tomography (CBCT) was not employed during the follow-up period. While CBCT would have allowed for a more precise, three-dimensional quantitative evaluation of periapical bone regeneration and root wall dimensions, in accordance with current position statements by the AAE and AAOMR [38], as well as the ESE [39], routine CBCT imaging is not indicated for the follow-up of endodontically treated teeth when the patient is completely asymptomatic and continuous periapical healing is clearly evident on conventional 2D radiographs. Therefore, the lack of 3D volumetric validation for hard tissue deposition remains an inherent limitation of this report.

## 4. Conclusions

The present case illustrates that in this particular instance, extraction may not be the only option for teeth with failed apexification complicated by severe structural defects. Through a sandwich-like three-layer configuration comprising biological sealing, mechanical isolation, and structural reinforcement, supplemented by minimally invasive disinfection and individualized occlusal management, the tooth achieved stable clinical and radiographic healing over 56 months. Although limited to a single case, this finding indicates that with careful individualized treatment planning, such complex cases previously considered as indications for extraction may still be salvageable.

## Data Availability

The original contributions presented in this study are included in the article. Further inquiries can be directed to the corresponding authors.
